# Supplementary material for: An analysis of humanitarian and health aid harmonisation over a decade (2011–2019) of the Syrian conflict
Source: BMJ Glob Health. 2024 Oct 21;9(10):e014687. doi: 10.1136/bmjgh-2023-014687 (PMC11499853; doi:10.1136/bmjgh-2023-014687)
Supplement: online supplemental file 1 [file bmjgh-9-10-s001.pdf]

Appendix 1

Structure of FGDs, Interviews, and Consultations

1. Focus Group Discussions (FGDs)

a. Overview: The FGDs aimed to gather in-depth qualitative insights from key stakeholders involved in the humanitarian and health response in Syria over the study period. The discussions were designed to complement the quantitative data and comprehensively understand local perspectives on aid harmonisation.

b. Participant Selection:

- **Sampling Methods:** Purposive sampling followed by snowballing techniques were used to ensure a diverse and representative group of participants.
- **Criteria for Selection:** Participants were selected based on their experience and involvement in the Syrian health and humanitarian responses. This included senior humanitarian workers from medical NGOs, INGOs, local authorities, technical institutions, and the Health Cluster based in Gaziantep.
- **Demographics:**
    - Total Invitees: 31
    - Participants: 25
    - Medical Background: 88%
    - Practicing Medical Profession Inside Syria: 20%
    - Gender: all participants were male (This reflects gender imbalance in the humanitarian sector in Syria; we sent invitations to all expected organisations with the suggested criteria; the agreed organisations named their representatives).
    - Locations: all participants are based in Turkey and Syria

c. Distribution of Participants:

- Ministry of Health in the Syrian Interim Government: 2
- Idlib Health Directorate: 2
- Health Information System Unit: 1
- Syrian Board of Medical Specialties: 1
- INGOs: 6
- NGOs: 12
- Health Cluster: 1

d. FGD Structure:

- **Number of FGDs:** Four
- **Group Composition:** Two sessions at a time, with an average of 12 participants per group.
- **Representation:** Ensured balanced stakeholder participation.
- **Format:** The discussions were structured but flexible, allowing for open-ended conversations facilitated by a moderator. Each session began with an introduction to the study's objectives, followed by a presentation of the quantitative results, guided

questions, and an open discussion to capture additional insights and participant feedback.

- **Timing:** The FGDs were conducted in August 2021 in Mersin, Türkiye.
- **Duration:** Each FGD session lasted approximately 2 to 2.5 hours.

e. Procedure:

- **Language:** Arabic
- **Roles:** Each group had a moderator and a note-taker.
- **Consent:** Verbal consent was obtained, with participants preferring discussions not to be recorded.

f. Discussion Guide:

- **Main Questions:**
    - To what extent do you agree with the quantitative findings regarding 1) pooled humanitarian and health aid against the crisis timeline and developing and fragile states; 2) aid fragmentation? Why?
    - How can aid harmonisation be improved further?
    - An open question: “Do you have anything to add to our discussions?”

g. Data Handling:

- **Note-Taking:** Detailed notes were taken by two designated note-takers.
- **Anonymisation:** Data was anonymised and aggregated to protect participant privacy.
- **Ethical Considerations:** Ethical consent and privacy protection measures were strictly followed.

2. Key Informant Interviews (KIIs)

a. Overview: The KIIs were conducted to explore policy implications from the perspectives of leading donors involved in Syria’s humanitarian and health response.

b. Participant Selection:

- **Criteria:** Representatives from four leading donor organisations engaged in Syria’s humanitarian and health responses.
- **Profile:** Participants included representatives involved in areas under various control regimes, including Syrian regime-controlled areas.
- **Demographics:**
    - Gender: 2 females and 2 meals
    - Locations: participants were based in neighbouring countries of Syria and Europe.

c. Interview Structure:

- **Number of Interviews:** Four
- **Duration:** Each KII session lasted approximately 50 – 60 minutes.
- **Timing:** Conducted in September and October 2021.

- **Format**: The discussions were structured but flexible, allowing for open-ended conversations facilitated by a moderator. Each interview began with an introduction to the study's objectives, followed by a presentation of the quantitative results, reflections on FGDs insights as needed, guided questions, and an open discussion to capture additional insights and participant feedback.

d. Procedure:

- **Language:** English
- **Recording:** Interviews were recorded and later transcribed.
- **Consent:** Written consent was obtained from all participants.

e. Interview Guide:

- **Main Questions:**
    - Validation of quantitative and FGDs results.
    - Policy implications of aid harmonisation.
    - Open question: Additional insights or comments.

f. Data Handling:

- **Transcription:** Interviews were transcribed and anonymised using unique identifiers.
- **Ethical Considerations:** Ethical consent and privacy protection were maintained.

3. Individual Consultations

a. Overview: Individual consultations aimed to gather expert opinions on early recovery requirements and the Aid Fund for Northern Syria (AFNS) role.

b. Participant Selection:

- **Criteria:** Experts in the humanitarian and development sectors in Syria.
- **Profile:** Participants included senior humanitarian experts and practitioners.
- **Demographics:**
    - Gender: 2 females and 1 meal
    - Locations: all participants are based in Europe.

c. Consultation Structure:

- **Number of Consultations:** Three
- **Duration:** consultations were conducted between January and August 2023.
- **Format:** we shared the final manuscripts with the consultants and asked them for written feedback focusing on two main areas: early recovery requirements and the role of ANFS.

d. Procedure:

- **Language:** English.
- **Consent:** Verbal consent to participate in the study and written consent to mention two of three consultants' names in the acknowledgement section were obtained.

e. Discussion Guide:

- **Main Topics:**
  - Early recovery requirements.
  - Role and effectiveness of AFNS.

f. Data Handling:

- **Note-Taking:** Detailed notes were taken.
- **Ethical Considerations:** Ethical consent and privacy protection measures were strictly followed.

4. Ethical Considerations

a. Consent Process:

- **FGDs and Consultations:** Verbal consent was obtained based on participant preference.
- **KIIs:** Written consent was obtained.
- **Participant Information:** Participants were informed about the study's purpose, data handling, and publication plans.

b. Data Privacy:

- **Anonymisation:** Data was anonymised to protect participant identities.
- **Storage:** Data was securely stored and handled in compliance with ethical standards.

5. Summary of Findings

a. Thematic Analysis:

- **Process:** Data from FGDs, KIIs, and consultations were thematically analysed.
- **Key Themes:** Themes are based on the study objectives and discussions within FGDs and KIIs

  - Trends in humanitarian and health pooled fund flows concerning the key conflict and crises indicators.
  - Health and humanitarian aid as pooled fund compared to other developing countries and fragile states.
  - Humanitarian and health aid fragmentation.
  - Impact of donor policies and coordination mechanisms.
  - Challenges in aid delivery.
  - Community and beneficiary perspectives.
  - Recommendations for improved aid effectiveness.

b. Integration with Quantitative Data:

- **Complementary Insights:** Qualitative findings provided contextual depth to the quantitative data, highlighting local and donor perspectives and nuanced understanding of aid harmonisation.

The end of Appendix 1
