# Supplementary material for: An analysis of humanitarian and health aid harmonisation over a decade (2011–2019) of the Syrian conflict
Source: BMJ Glob Health. 2024 Oct 21;9(10):e014687. doi: 10.1136/bmjgh-2023-014687 (PMC11499853; doi:10.1136/bmjgh-2023-014687)
Supplement: online supplemental file 2 [file bmjgh-9-10-s002.docx]

**Reflexivity Statement**

Many authors of this paper are Syrians who have been involved in the health and humanitarian responses to the Syrian humanitarian crisis since 2011. The lead author Dr Munzer Alkhalil co-founded many health quasi-governmental institutions in northwest Syria, including the Idlib Health Directorate (IHD); he was the head of IHD between 2011 and 2020. As such, Syrian authors bring direct experience and understanding of humanitarian response and mechanisms in Syria. International academic authors also guaranteed the balance between local and global perspectives and practical and theoretical dimensions. Finally, this paper is the third one related to this research consortium's series on aid effectiveness, following previous works on alignment and aid displacement.
